# Supplementary material for: Controlling noise with self-organized resetting
Source: Commun Phys. 2025 Feb 12;8(1):63. doi: 10.1038/s42005-025-01985-7 (PMC11813803; doi:10.1038/s42005-025-01985-7)
Supplement: Supplementary file 2 — Supplementary Information [file 42005_2025_1985_MOESM2_ESM.pdf]

Controlling noise with self-organized resetting: Supplementary Information

FELIX J. MEIGEL AND STEFFEN RULANDS

SUPPLEMENTARY NOTE 1: DEFINITION OF THE RESETTING AND THE DIFFUSION OPERATOR

As introduced in the main text of the manuscript, we consider N particles described by their positions $X_i \in \mathbb{R}$ in one spatial dimension, which undergo Brownian motion with diffusion constant D and which are subject to cooperative resetting dynamics. To describe the collective behavior of this system, we study the single-particle probability density, $p(x,t)$. The time evolution of $p(x,t)$ is governed by two processes: the effect of Brownian motion, which depends on the single-particle density $p(x,t)$, and the pair-wise resetting, which depends on the two-particle density $p_2(x,x',t)$. We define the diffusion operator at position $\tilde{x}$ as

$$\hat{\mathcal{L}}[p(x,t)]\big|_{x=\tilde{x}} = D\frac{\partial^2}{\partial x^2}p(\tilde{x},t) \tag{S1}$$

which is the standard expression for the diffusion flux. For the resetting operator, we compute the rate of a particle being added at position $\tilde{x}$ and subtract the rate of a particle being removed at position $\tilde{x}$ due to cooperative resetting,

$$\begin{aligned}\hat{\mathcal{R}}[p_2(x,x',t)]\big|_{x=\tilde{x}} =& \frac{1}{2}\int_{-\infty}^{\infty}\int_{-\infty}^{\infty} \mathrm{d}x\,\mathrm{d}x'\, p_2(x,x',t)\mu(x-x')\delta\left(\tilde{x}-\frac{x+x'}{2}\right)\\ &-\int_{-\infty}^{\infty}\mathrm{d}x'\, p_2(\tilde{x},x',t)\mu(\tilde{x}-x').\end{aligned} \tag{S2}$$

SUPPLEMENTARY NOTE 2: FLUX FORMULATION OF THE RESETTING AND THE DIFFUSION OPERATOR

Accounting for the fact that resetting conserves the total number of particles, instead of referring to source and sink terms, we consider the displacement of particles. This allows us to rephrase the resetting operator as a derivative of a flux

$$\hat{\mathcal{R}}[p_2(x,x',t)]\big|_{x=\tilde{x}} = -\frac{\partial}{\partial x}\hat{J}_{\mathcal{R}}[p_2(x,x',t)]\Big|_{x=\tilde{x}} \tag{S3}$$

where the flux is

$$\hat{J}_{\mathcal{R}}[p_2(x,x',t)]\big|_{x=\tilde{x}} = \frac{1}{2}\int_{-\infty}^{\infty}\int_{-\infty}^{\infty}\mathrm{d}x\,\mathrm{d}x'\, p_2(x,x',t)\mu(x-x')\Theta((x-\tilde{x})(\tilde{x}-x))\mathrm{sgn}\left(\frac{x+x'}{2}-\tilde{x}\right). \tag{S4}$$

Analogously, the diffusion processes can be rephrased as the derivative of a diffusion flux, where the diffusion flux is

$$\hat{J}_{\mathcal{L}}[p(x,t)]\big|_{x=\tilde{x}} = -D\frac{\partial}{\partial x}p(x,t). \tag{S5}$$

SUPPLEMENTARY NOTE 3: APPROXIMATION OF THE RESETTING FLUX

We approximate the resetting flux by making use of a mean-field approximation such that $p_2(x,x',t) = p(x,t)p(x',t)$. We define two integration sets by making the definition of the sign function explicit,

$$\begin{aligned}I_+(\tilde{x}) &= \left\{(x,x')\,|\,x<\tilde{x}\wedge\frac{x'+x}{2}>\tilde{x}\right\}\\ I_-(\tilde{x}) &= \left\{(x,x')\,|\,x>\tilde{x}\wedge\frac{x'+x}{2}<\tilde{x}\right\}.\end{aligned} \tag{S6}$$

Furthermore, we perform a transformation to spherical coordinates, such that

$$\begin{aligned} x &= \cos(\theta) r + \tilde{x} \\ x' &= \sin(\theta) r + \tilde{x}. \end{aligned} \tag{S7}$$

The double integral then simplifies to

$$\hat{J}_{\mathcal{L}}[p(x,t)]\big|_{x=\tilde{x}} = \int_{\pi/2}^{\frac{3\pi}{4}} \mathrm{d}\theta \int_0^\infty \mathrm{d}r\, r\, \mu(r(\cos(\theta)-\sin(\theta)))\, [f(\cos(\theta)r+\tilde{x}) f(\sin(\theta)r+\tilde{x}) \\ - f(-\cos(\theta)r+\tilde{x}) f(-\sin(\theta)r+\tilde{x})]\,. \tag{S8}$$

We find that this transformation allows to extract the intrinsic symmetries of the integral. We define

$$g(r,\theta,\tilde{x},t) = \mu(r(\cos(\theta)-\sin(\theta)))\, (f(\cos(\theta)r+\tilde{x}) f(\sin(\theta)r+\tilde{x}) \\ - f(-\cos(\theta)r+\tilde{x}) f(-\sin(\theta)r+\tilde{x}))\,, \tag{S9}$$

as the integrand. The line integral vanishes along the axis $\theta = 3\pi/4$ by construction. Furthermore, the line integral $\int_0^\infty \mathrm{d}\, r g(r,\theta,\tilde{x},t)$ is maximal along the axis $\theta = \pi/2$ and decays monotonically in $\theta \in [\pi/2, 3\pi/4]$ if $p(x+x',t)^2 > p(x,t)p(x+2x',t)$ for all $x, x' \in \mathbb{R}$.

We next perform a first-order expansion over the arc integral as we take the limit $r \to \infty$, which can be visualized as a triangulation of the integral. We find that the arc integral is approximated by

$$I_\theta(r,\tilde{x},t) = \frac{1}{2}\mu(r) \left| \frac{p(\tilde{x},t)}{r p'(\tilde{x},t)} \right| [p(\tilde{x}+r,t) - p(\tilde{x}-r,t)]\, p(\tilde{x},t) + \mathcal{O}(r^{-(\alpha+1)}). \tag{S10}$$

The integral can be read as the area of a triangle of height $h = p(\tilde{x}+r,t)p(\tilde{x},t)$ and base $\mu(r) \left| \frac{p(\tilde{x},t)}{r p'(\tilde{x},t)} \right|$. Here, we find that the inverse normalized gradient arises from a linearization at $\theta = \pi/2$ along the arc as we solve a function of the form $0 = m + b\theta$.

Next, we account for the radial integration and the back transformation of the variables. To this end, we find that the approximation of the arc integral to leading order in $1/r$ is admissible if we consider the flux in the tail of $p(x)$, which is equivalent to taking the limit $|x| \to \infty$. With this, we find that the double integral simplifies to

$$\hat{J}_{\mathcal{R}}[p_2(x,x',t)]\big|_{x=\tilde{x}} \approx \frac{1}{2} \frac{\mu_0}{|\tilde{x}|^\alpha} p(\tilde{x}) \left| \frac{p(\tilde{x})}{p'(\tilde{x})} \right|. \tag{S11}$$

Note, that without performing the radial integral, we derive at an equation for the time evolution of $p(x,t)$ which is reminiscent of a McKean-Valsov equation, similar to mean-field limit of the Dean-Kawasaki equation [1]:

$$\frac{\partial}{\partial t} p(x,t) = D \frac{\partial^2}{\partial x^2} p(x,t) + \frac{\partial}{\partial x} \left[p(x,t) \left| \frac{p(\tilde{x},t)}{p'(\tilde{x},t)} \right| \int_{-\infty}^{\infty} \mathrm{d}x' p(x',t) \mathrm{sign}(x-x') \mu(x-x') \right] \tag{S12}$$

Yet, in contrast to the Dean-Kawasaki mean-field limit we get an additional correction factor in front of the interaction integral.

SUPPLEMENTARY NOTE 4: HIGHER-ORDER APPROXIMATION FOR ALGEBRAICALLY DECAYING PROBABILITY DENSITIES

From Eq. (4) in the main text we find that for quadratically decaying resetting interactions the steady state probability densities show an algebraic decay with $p_s(\chi) \propto \chi^{-\sqrt{\varrho}}$ for $|\chi| \to \infty$. We validated this result for $\varrho \gg 1$ numerically (Fig. 2**b** in the main text). Yet, the condition $p(x+x',t)^2 > p(x,t)p(x+2x',t)$ for all $x, x' \in \mathbb{R}$ is not fulfilled for algebraically decaying probability distribution. As a consequence, $g(r,\theta,\tilde{x},t)$ is not granted to be a monotonic function on the interval $\theta \in [pi/2, 3\pi/4]$. In particular in the region, where the probability distribution can be well approximated by an algebraic decay, $g(r,\theta,\tilde{x},t)$ obtains both negative and positive values. Nonetheless, the approximation still works well for the region close to the center of the distribution around x_0, which is dominating the resetting flux.

To refine the approximation, we define two regions. Given the interaction length scale δ_0, we define a region $[x_0 - 2\delta_0, x_0 + 2\delta_0]$ as the center of the distribution and the remaining parts of the distribution as the tail region of the distribution. We find that due to the non-monotinicity of $p(x) \propto x^{-a}$ the region $r > \tilde{x} + 2\delta 2$ is inflicted with negative values of $g(r, \theta, \tilde{x}, t)$ in the interval $\theta \in [pi/2, 3\pi/4]$. Here, we defined the exponent of the decaying probability distribution to be a. Effectively, the arc integral $I_\theta(r, \tilde{x}, t)$ vanishes in the tail region. We account for this by employing a factor $\Lambda(a) = 1 - \int_{2\delta}^{\infty} \mathrm{d}x p(x) - \int_{-\infty}^{-2\delta}$ which modifies equation Eq. (4) in the main text as

$$\varrho\, \Lambda(a)\, p_{\mathrm{s}}(\chi) \left| \frac{p_{\mathrm{s}}(\chi)}{p_{\mathrm{s}}'(\chi)} \right| |\chi|^{-\alpha} \stackrel{!}{=} p_{\mathrm{s}}'(\chi)\,. \tag{S13}$$

As a consequence, the steady state probability distribution needs to solve the equation $\varrho\, \Lambda(a) = a^2$. We find that $\Lambda(a)$ is well approximated by the analytical function $\Lambda(a) \approx= 1 - (2^{(1-a)} a \sin(\pi/a))/((-1+a)\pi)$. Making use of this function, we find that the consistency equation only yields solutions $a(\varrho)$ if $\sqrt{\varrho} > 2.3$ This gives a refined approximation for the critical rescaled density in line with the numerical observation in Fig. 2**d** in the main text.

SUPPLEMENTARY REFERENCES

1. N. Martzel and C. Aslangul, "Mean-field treatment of the many-body Fokker-Planck equation," J. Phys. A: Math. Gen. **34**, 11225 (2001).
